# Editorial: Electromyography (EMG) Techniques for the Assessment and Rehabilitation of Motor Impairment Following Stroke

**DOI:** 10.3389/fneur.2018.01122

**Published:** 2018-12-18

**Authors:** Cliff S. Klein, Sheng Li, Xiaogang Hu, Xiaoyan Li

**Affiliations:** ^1^Guangdong Work Injury Rehabilitation Center, Guangzhou, China; ^2^University of Texas Health Science Center at Houston, and TIRR Memorial Hermann Research Center, Houston, TX, United States; ^3^Joint Department of Biomedical Engineering, University of North Carolina at Chapel Hill and North Carolina State University, Raleigh, NC, United States

**Keywords:** electromyography, MUSC, motor, rehabilitation, stroke

The nineteen papers of the research topic Electromyography (EMG) Techniques for the Assessment and Rehabilitation of Motor Impairment Following Stroke highlight a variety of ways that EMG may be used to better understand and treat stroke-induced brain damage. Seven papers addressed the impact of weekly training on EMG properties and function post-stroke, and one paper examined the effect of a robotic exoskeleton on gait during a single training session (**Exercise/therapy interventions)**. Six of the seven training studies were concerned with upper limb function (one of which also assessed corticomuscular coupling), and one examined the effect of foot drop stimulator training. The six upper limb studies used a variety of training modalities including Wii-based upper limb therapy (two papers from one group), EMG-driven robotic devices with or without neuromuscular electrical stimulation (NMES) (three papers), and traditional physical/occupational therapy (one paper).

Another seven papers were focused on using EMG to examine motor impairment after stroke (**Mechanisms of motor impairment)**. These included one study that addressed coupling between the index finger and thumb, whereas another addressed upper limb synergies during reaching. One paper examined EMG co-contraction during gait, and one addressed gait EMG during obstacle crossing. One group examined reticulospinal pathways during elbow flexor activity using startling acoustic stimulation. Another studied masticatory muscle activity following brainstem stroke. Finally, one group addressed coupling between the electroencephalogram (EEG) and EMG signals during upper limb movements.

Four studies used novel EMG processing techniques to study motor control and impairment post-stroke **(Novel EMG processing techniques)**. These included new approaches to intramuscular EMG decomposition, coherence of motor unit firing patterns from surface EMG, clustering index analysis of surface EMG, and pattern recognition from high density surface EMG.

## Exercise/therapy Interventions

A number of studies examined the effects of an exercise/therapy program, or a single exercise session, on EMG and motor function. Some also addressed the associated cortical plasticity.

### Upper Limb

Many addressed the effect of exercise/therapy on upper limb muscle activation properties. Hesam-Shariati et al. examined changes in upper limb EMG activity resulting from the standardized 14-day Wii-based Movement Therapy program (i.e., Wii-tennis, golf, baseball) in chronic stroke survivors. They found that training lead to different patterns of EMG changes that were related to the level of motor deficit. In their companion paper Hesam-Shariati et al. they quantified muscle synergies *during* therapy (Wii baseball swing) based on EMG activity of the affected arm muscles using a non-negative matrix factorization algorithm. They were able to identify differences in the number of muscle synergies used by patients as a function of the level of motor deficit.

Device-assisted interventions offer a way to study and train patients with more severe limb impairment. In a case study, Lu et al. used forearm EMG signals to detect a stroke survivor's motion intent, and then used the EMG to drive a hand exoskeleton to assist with finger motion in real time. After 10-weeks of robot-assisted hand therapy, the patient showed improved grip strength and hand function. The results demonstrate the feasibility of robot-assisted training driven by myoelectric pattern recognition in chronic stroke survivors.

After a stroke, it is critically important to start rehabilitation early to take advantage of the highly plastic period of the neural system. In a pilot randomized control trial, Qian et al. evaluated the effects of 1 month (20 sessions) of EMG-driven NMES combined with robotic assistance, targeting the elbow, wrist, and fingers of subacute stroke survivors. EMG parameters, including the co-contraction index and the activation level of targeted muscles were used to monitor the muscle coordination patterns. They found that the NMES combined with robotic training could achieve higher motor outcomes at the distal joints and more effective reduction in muscle tone than traditional therapy.

Some investigators also addressed training-related cortical plasticity and corticomuscular coupling. Wilkins et al. found EMG-driven NMES task-specific arm/hand training (7 weeks) improved hand opening and functional use in chronic stroke survivors with moderate to severe motor impairments. Functional improvement was paralleled with functional reorganization in the ipsilesional primary sensorimotor cortex. The neural plastic reorganization after functional improvement was also seen with strengthened corticomuscular coupling. In a case study of a subacute stroke subject, Zheng et al. evaluated corticomuscular coupling between EEG and EMG (biceps) signals during elbow flexion before and after 1 month of regular physical and occupational therapy. Corticomuscular coherence was increased in the affected limb with functional improvement, but not in the non-affected limb. These results exemplify that stroke survivors with severe motor impairments may still have the potential to improve hand function if appropriate interventions are used to induce neural plasticity.

### Lower Limb

Pilkar et al. used different EMG-based indices to quantify the effects of a foot drop stimulator on muscle activation during gait over a 6 month period of community walking. A wavelet-based time-frequency analysis approach was used to quantify activation changes of multiple ankle muscles in chronic stroke survivors. The findings suggest alterations in motor unit recruitment strategies after foot drop stimulator use. The outcomes establish the efficacy of a foot drop stimulator as a rehabilitation intervention that may promote motor recovery in addition to reducing foot drop.

Quantitative and continuous monitoring of muscle activation is necessary to adjust training protocols in a timely manner. Androwis et al. used novel EMG analysis (Burst Duration Similarity Index) to quantify the intensity and timing of muscle activation during a single session of robotic gait therapy in acute stroke survivors. The authors showed that a robotic exoskeleton can reduce the soleus and rectus femoris muscle activity in the affected limb during stance phase, and can also improve the timing of muscle activation in the affected limb.

### Mechanisms of Motor Impairment

Surface EMG together with other signals recorded peripherally or centrally provides a means to assess mechanisms of motor impairment. Jones and Kamper studied the coupling of the index finger and thumb during close-open pinching motions in chronic stroke survivors. A Cable-Actuated Finger Exoskeleton was used to perturb joints of the index finger during pinching motions, while finger/thumb muscle surface EMG and finger kinematics were recorded. They found that involuntary finger-thumb coupling was present during the dynamic pinching task, with perturbation of the index finger impacting thumb activity. This finding reveals a potential mechanism to improve hand mobility following stroke. Li et al. analyzed motor synergies during arm reaching based on surface EMG recordings from multiple muscles and correlated with reaching kinematics. They were able to detect task-specific deficits in reaching movements after stroke

Ma et al. studied lower limb muscle activity during obstacle crossing using surface EMG in chronic stroke survivors. EMG activity of the leading limb during the swing phase was larger in all muscles in the stroke compared to the control group, and TA activity increased with obstacle height in both groups. Co-contraction between agonist-antagonist muscle pairs was larger in the stroke group in the leading/ trailing limb during certain phases. The authors suggested that the greater muscle activation during obstacle crossing following stroke may have a negative impact on balance.

It remains unclear whether co-contraction of agonist-antagonist muscles is excessive and impacts gait significantly following stroke. In chronic stroke survivors, Banks et al. quantified surface EMG co-contraction of agonist-antagonist muscle pairs in three ways (no normalization, normalized to the maximal EMG of the gait cycle, normalized to the M-wave) and determined their association with gait impairment during treadmill walking. Co-contraction during the terminal stance phase was not different between healthy controls and the stroke subjects, regardless of the normalization method. Normalization also did not impact the ability to resolve group differences. Furthermore, the correlation between stance phase co-contraction and walking speed was modest. Pathological co-contraction may not be a primary factor contributing to impaired gait in most stroke survivors. The authors suggest other approaches that account for timing and amplitude components of the EMG (i.e., muscle synergy analysis) may better capture the relevant deficits.

The coupling strength between the EEG and EMG signals during motion is instructive in assessing motor function. Gao et al. studied subacute stroke patients completing tasks such as hand gripping and elbow bending. Stroke subjects demonstrated greater strength in the bi-directional corticomuscular coupling between the EEG and EMG signals. Such changes suggest a compensational strategy after the brain lesion.

It is difficult to assess activities of brainstem nuclei *in vivo* even with the most advanced neuroimaging techniques. Startling acoustic stimulation is known to stimulate the reticulospinal pathways, thus allowing the opportunity to assess the role of brainstem motor system indirectly. Li et al. analyzed changes in EMG and force in response to startling acoustic stimulation during isometric elbow flexion in stroke survivors and healthy controls. They reported that the sound-induced force and EMG increase in stroke survivors was not significantly different from those in healthy controls. As such, there results suggest that the reticulospinal projections do not increase their contributions to muscle strength in stroke. Jian et al. analyzed surface EMG signals of bilateral masticatory muscles in stroke survivors after brainstem stroke using multiple EMG parameters. In addition to expected differences between muscles and sides, they did not observe the head position effect on muscle activation on both sides. These are valuable information as the results could advance the understanding whether head positions alter chewing and swallowing activities in stroke survivors.

### Novel EMG Processing Techniques

Examining motor unit discharge and recruitment patterns post-stroke can disclose valuable information pertaining to impaired spinal versus supraspinal motor control. EMG decomposition into constituent motor unit action potential (MUAP) trains, however, is challenging with severe superposition of multiple MUAPs. Ren et al. developed a new intramuscular EMG decomposition technique to improve the accuracy of EMG decomposition with interference patterns. The technique was implemented by using six stages of analysis including feature extraction, clustering, refinement of the classification, and splitting of the superimposed MUAPs. A high accuracy of MUAP detection was reported in 8 subacute stroke survivors (88%) and 20 healthy control participants (94%).

Dai et al. quantified the different types of connectivity in the spinal networks and changes in their relative contributions after a stroke. By comparing the coherence of motor unit firing pattern across different isometric contractions, they identified significant changes in coherence in three frequency bands: delta (1–4 Hz), alpha (8–12 Hz), and beta (15–30 Hz) in the paretic hand muscles. These changes reflect increased common synaptic inputs in the subcortical pathway and provide evidence on different origins of impaired muscle activation in stroke.

To further differentiate neurogenic and myopathic changes in the muscle, Tang et al. applied clustering index analysis to examine surface EMG in the distal and proximal muscles of the upper limb from 12 stroke survivors. They observed abnormally high or low clustering index values in the paretic muscles compared to healthy controls. This finding may indicate that both neurogenic and myopathic changes may occur in paretic muscles.

Selection of appropriate features from surface EMG is essential for development of highly effective pattern recognition algorithms in the EMG-controlled devices. Wang et al. developed a novel pattern recognition technique for precise discrimination of 20 hand/upper limb functional movements in stroke survivors. Specifically, they applied wavelet packet to extract the neural control features and used the Fisher's class separability index and the sequential feedforward selection analyses to select appropriate channels in high density surface EMG. Such implementation can facilitate use of surface EMG control in stroke rehabilitation.

## Author Contributions

All authors made an equal contribution to the work, and approved it for publication.

### Conflict of Interest Statement

The authors declare that the research was conducted in the absence of any commercial or financial relationships that could be construed as a potential conflict of interest.

